# Scoring System for Lesions Induced by Different Strains of Newcastle Disease Virus in Chicken

**DOI:** 10.1155/2018/9296520

**Published:** 2018-12-09

**Authors:** Elawad A. Hussein, M. Hair-Bejo, Lawan Adamu, A. R. Omar, Siti S. Arshad, Elmutaz A. Awad, I. Aini

**Affiliations:** ^1^Department of Veterinary Pathology and Microbiology, Faculty of Veterinary Medicine, Universiti Putra Malaysia, 43400 UPM Serdang, Selangor, Malaysia; ^2^Institute of Bioscience, Universiti Putra Malaysia, 43400 UPM Serdang, Selangor, Malaysia; ^3^Department of Veterinary Clinical Studies, Faculty of Veterinary Medicine, Universiti Putra Malaysia, 43400 UPM Serdang, Selangor, Malaysia; ^4^Institute of Tropical Agriculture and Food Security, Universiti Putra Malaysia, 43400 UPM Serdang, Selangor, Malaysia

## Abstract

Newcastle disease virus strains are velogenic, mesogenic, and lentogenic. This study aims to design a scoring system for lesions induced by different strains of Newcastle disease virus in chicken. Three experiments were conducted. In experiments 1 and 2, chickens were divided into infected and control groups. Infected groups of experiments 1 and 2 consisted of 6 and 24 specific pathogen-free (SPF) chickens, respectively. Control groups in experiments 1 and 2 consisted of 6 and 15 SPF chickens, respectively. In infected groups, infection was induced by intranasal administration of 10^5^ 50% EID_50_/0.1 mL of velogenic Newcastle disease virus strain (vNDV). Infected chickens in experiment 1 were euthanised by cervical dislocation on days 3, 6, and 7 postinoculation (pi). Infected chickens in experiment 2 were euthanised at hours (hrs) 2, 4, 6, 12 and days 1, 2, 4, and 6 pi. Chickens of the control group in experiment 1 were euthanised on days 3 and 7 pi, whereas control group chickens in experiment 2 were euthanised on days 0, 1, 2, 4, and 6 pi. Then in experiment 3, 15 SPF chickens were divided into three groups; in the first group, 5 SPF chickens were infected with vNDV, in the second group, 5 SPF chickens were infected with lentogenic NDV (lNDV) (10^3.0^ EID_50_/0.1 mL), and the third group was kept without infection as a control group. Chickens were euthanised on day 5 pi. In all previous experiments, tissues of brain, trachea, lung, caecal tonsil, liver, kidney, spleen, heart, proventriculus, intestine, and thymus were collected, fixed in 10% buffered formalin, embedded in paraffin, and sectioned. HS staining was applied. Tissues were examined under light microscope and changes were recorded. A scoring system was designed for lesions induced by different strains of NDV and, accordingly, lesions were scored. The scoring system was found helpful in the evaluation of disease severity.

## 1. Introduction

Newcastle disease virus (NDV) can be grouped into five pathotypes with respect to tissue tropism and clinical signs, that is to say, (a) viscerotropic velogenic pathotype and (b) neurotropic velogenic pathotype. Both viscerotropic and neurotropic pathotypes cause high mortality rate accompanied by intestinal lesions or nervous signs; (c) mesogenic pathotype causes a low mortality rate and respiratory and nervous signs; (d) lentogenic pathotype is the causative agent of clinically mild or unapparent infections of respiratory tract; and eventually (e) asymptomatic pathotype and its reflection on chicks are unapparent intestinal infections [1]. Based on the severity of infection, there is also another classification of Newcastle disease virus. Here, there are velogenic, mesogenic, and lentogenic strains of ND virus (NDV). In other words, the isolates which do not manifest clinical picture are considered as lentogenic, the isolates of intermediate virulence are called mesogenic, and virulent isolates of high mortality rate are termed velogenic [2–4].

Due to the different tissue tropisms of different pathotypes and variant strains and too many tissues invaded by the virus, it is important to design a comprehensive scoring system to evaluate the virus virulence, to evaluate the disease severity with respect to lesions induced, and even to evaluate the impact of the vaccine strains whether it is delicate or drastic in enhancement of tissue changes and injuries. Previously there were some attempts for designing some scoring systems, but they were applicable to a limited number of tissues and sometimes to limited grades [5, 6]. The objectives of this study were to design a scoring system for the lesions induced by different strains of Newcastle disease virus on 15 different tissues and to apply the system on different tissues infected by velogenic and lentogenic strains of NDV.

## 2. Materials and Methods

### 2.1. Ethical Approval

The study was carried out in accordance with the Institutional Animal Care and Use Committee (IACUC), Faculty of Veterinary Medicine, Universiti Putra Malaysia (UPM).

### 2.2. Chickens

Specific pathogen-free chickens (SPF) were used for 3 experiments. At the onset of every experiment, the chickens were 4 weeks old. The breed was white leghorn.

### 2.3. Viruses

For the first, second, and third experiments, vNDV strain with a dosage of 0.1 mL 10^5^ EID50 /0.1 mL of AF2240 isolate was inoculated, while V4 isolate was inoculated as a lNDV (10^3.0^ EID_50_/0.1 mL) in one group of the third experiment.

### 2.4. Experimental Design

#### 2.4.1. Experiment 1

Six SPF chickens were infected with vNDV by intranasal administration of 0.1 mL 10^5^ EID50 /0.1 mL of AF2240 isolate. Every chicken was inoculated with 0.1 mL. A separate group consisting of 6 chickens was reserved without infection as a group of control. Chickens were provided with water and feed* ad libitum*. Chickens were monitored every day for record of the clinical signs and gross lesions. Three chickens from the infected group were sacrificed by cervical dislocation on day 3 pi. The tissues of trachea, spleen, brain, lung, liver, bursa of Fabricius, proventriculus, and caecal tonsils were collected and fixed in 10% buffered formalin. The tissues of one dead chicken on day 6 pi were collected and fixed in 10% buffered formalin. Two chickens from the infected group were sacrificed on day 7 pi and so were tissues of the control group. Formalin-fixed paraffin-embedded tissues were prepared. Tissues were sectioned, stained with HE, and examined under light microscopy, and changes were recorded.

#### 2.4.2. Experiment 2

Twenty four SPF chickens were infected with ND by intranasal administration of 10^5^ EID50/0.1 mL of vND AF2240 isolate. A second group of 15 SPF chickens was kept as a control. Infected chickens were sacrificed by cervical dislocation at hrs 2, 4, 6, 12, days 1, 2, 4, and 6 pi. Control group chickens were sacrificed on days 0, 1, 2, 4, and 6 pi. For sampling, the tissues of the brain, trachea, lung, caecal tonsil, liver, kidney, spleen, heart, proventriculus, intestine, and thymus were fixed in 10% buffered formalin. Formalin-fixed paraffin-embedded tissues were prepared. Tissues were sectioned, stained with HE, and examined under light microscopy, and changes were recorded.

#### 2.4.3. Experiment 3

Five SPF chickens were infected with ND by intranasal administration of 10^5^ EID50/0.1 mL of AF2240 isolate which was vNDV. A second group of 5 SPF chickens was infected with V4 isolate (lNDV) (10^3.0^ EID_50_/0.1 mL) which was lNDV. A third group of 5 SPF chickens was kept as a control group without infection. All chickens were sacrificed by cervical dislocation on day 5 pi. For necropsy, the tissues of brain, trachea, caecal tonsil, liver, spleen, bursa of Fabricius, proventriculus, and thymus were fixed in 10% buffered formalin. Formalin-fixed paraffin-embedded tissues were prepared. Tissues were sectioned, stained with HE, and examined under light microscopy, and changes were recorded.

### 2.5. Scoring System for Infected Tissues

Scoring system for the lesions of NDV-infected-tissues was modified from the criteria described in some previous studies [5–7]. Briefly, in the trachea, larynx, bursa of Fabricius, spleen, and kidney, lesions were scored in 6 grades as 0 (normal), 1 (mild), 2 (mild to moderate), 3 (moderate), 4 (moderate to severe), and 5 (severe). The procedure to score lesions in tissue was described by Gaweco [8]. Five random optical fields were examined and scored, and then mean of five fields was calculated. Mean for 3 tissues ± standard error (SEM) was determined using SPSS.

*Trachea and larynx.* They were as follows: 0 = normal; 1 = hyperemia and inflammatory cells infiltration; 2 = hyperemia, inflammatory cells infiltration, and edema; 3 = hyperemia, inflammatory cells infiltration, edema and deciliation; 4 = slight hyperplasia and deciliation; 5 = haemorrhagic patches, desquamation and hyperplasia.

*Bursa of Fabricius.* It was as follows: 0 = normal; 1 = scattered follicles with mild necrosis; 2 = follicles suffering from moderate and generalized lymphoid depletion or even sometimes severe lymphoid depletion; 3 = over 50% of follicles suffering from severe lymphoid depletion; 4 = cysts in the follicles with scattered lymphocytes, increase in the size of connective tissue, thickening and folded epithelium; 5 = complete disruption of the follicular physique and increase in fibroplasia.

*Spleen.* It was as follows: 0 = normal; 1 = mild hyperplasia or hypertrophy in the ellipsoids; 2 = proliferative lymphoid follicles; 3 = degeneration in a mild focus form and numerous lymphoid follicles in an active form; 4 = necrosis in a disseminated focal manner and lymphoid follicles which were moderately active; 5 = necrosis in a diffuse and disseminated form and lymphoid follicles in a very active state.

*Kidney.* It was as follows: 0 = normal; 1 =mild accumulation of inflammatory cells in renal interstitium; 2 = inflammatory cells in association with foci of degeneration in the tubules; 3 = inflammatory cells in association with foci of degeneration and necrosis in the tubules; 4 = inflammatory cells beside degeneration and necrosis of the epithelium of medullary tubules; 5 = hyaline casts accompanied by interstitial nephritis.

In the thymus, proventriculus, Harderian gland, and heart, the lesions severity was scored in 5 grades as 0 (normal), 1 (mild), 2 (mild to moderate), 3 (moderate), and 4 (moderate to severe).

*Thymus.* It was as follows: 0 = normal; 1 = few vacuoles in the cortex; 2 = greater number of vacuoles in the cortex beside infiltration of the heterophils; 3 = marked cortical reduction with some round aggregations of cellular debris and pyknotic nuclei in the cortex; 4 = drastic atrophy in the cortex of the thymus.

*Proventriculus.* It was as follows: 0 = normal; 1 = mild epithelial cell degeneration and necrosis with heterophils; 2 = extensive epithelial cell degeneration and necrosis accompanied by mononuclear cell infiltration; 3 = destruction of the lymphoid areas and often fibrin was present; 4 = destroyed lymphoid areas with some haemorrhage.

*Harderian gland.* It was as follows: 0 = Normal; 1 = slight lymphocyte infiltration; 2 = heavy lymphocyte infiltration; 3 = little development of the lymphoid follicles; 4 = excessive development of the lymphoid follicles.

*Heart.* It was as follows: 0 = normal; 1 = infiltration of mononuclear Inflammatory cells; 2 = degeneration of myocardial fibres; 3 = myocardial fibres started to disrupt; 4 = disruption of cardiac myofibres and macrophages accumulation in the myocardium.

In the brain, caecal tonsil, liver, bone marrow, lung, and intestine, the severity of lesions was scored in 4 grades as 0 (normal), 1 (mild), 2 (moderate), and 3 (severe).

*Brain.* It was as follows: 0 = normal; 1 = perivascular infiltration of mononuclear cells; 2 = endothelial hypertrophy and neuronal necrosis; 3 = spongy change, gliosis, and haemorrhage.

*Caecal tonsil.* It was as follows: 0 = normal; 1 = very few proliferative lymphoid follicles in a section; 2 = many active lymphoid follicles; 3 = active lymphoid follicles considerably disseminated or focal necrosis.

*Liver.* It was as follows: 0 = normal; 1 = inflammatory cells infiltration; 2 = inflammatory cells infiltration accompanied by hepatocellular degeneration and necrosis; 3 = severe hepatocellular degeneration and necrosis.

*Bone marrow.* It was as follows: 0 = Normal; 1 = degeneration of endothelial cells; 2 = moderate degeneration and necrosis of lymphoid and granulocytic cells, lymphoid depletion and haemorrhage; 3 = severe degeneration and necrosis of the lymphoid and granulocytic cells.

*Lung.* It was as follows: 0 = normal; 1 = inflammatory cells infiltration in the air capillaries; 2 = inflammatory cells infiltration, haemorrhage and exudate into the secondary bronchi; 3 = hypertrophy of the tertiary bronchial epithelium and interstitial oedema.

*Intestine.* It was as follows: 0 = normal; 1 = mild to moderate infiltration of lymphocytic cells; 2 = moderate degeneration of the epithelial cell beside infiltration of mononuclear inflammatory cells usually in submucosal areas; 3 = haemorrhagic foci accompanied by necrosis in the mucosal lymphoid tissue.

## 3. Results

### 3.1. Experiment 1

**Figure 1:**
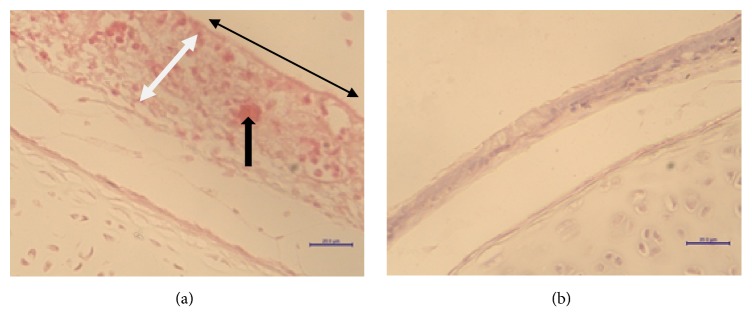
Trachea of SPF chickens. (a) Day 3 pi of vNDV. Hyperaemia (black arrow), hyperplasia (white double arrow), and deciliation (double arrow in black), lesion scoring of 4.00.Scale bar = 20 *μ*m. (b) Control group, lesion scoring of 0. Scale bar = 20 *μ*m. HE.

**Figure 2:**
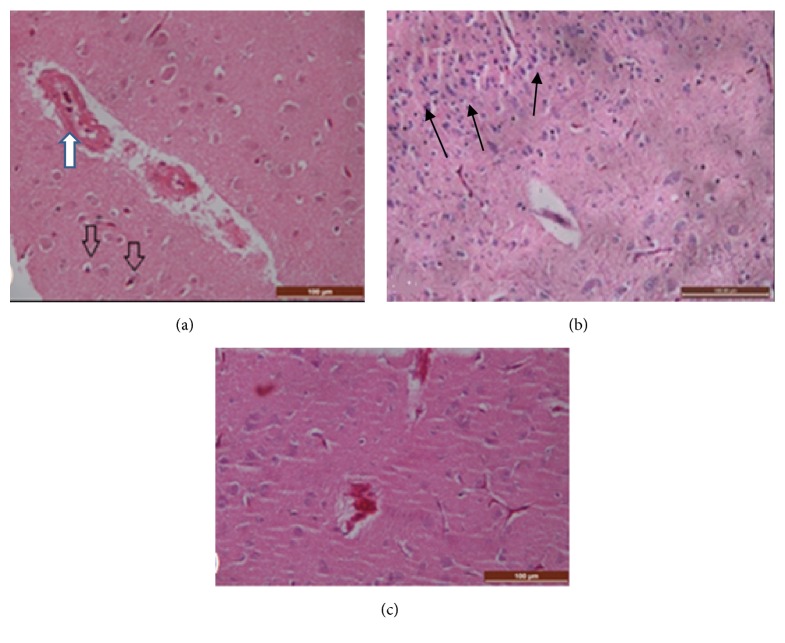
Brain of SPF chickens. (a) Day 5 pi of vNDV, endothelial hypertrophy (white arrow) and neuronal necrosis (black arrow). (b) Day 4 pi of vNDV, gliosis (arrow). (c) Control group, lesion scoring of 0. HE, scale bar = 100 *μ*m.

In the infected group of trachea, bursa of Fabricius, and spleen tissues which had scoring system of 6 grade (0-5), the lesions scoring in the trachea, on days 3, 6, and 7 pi, was 5.00 ± 0.00, 5.00 and 5.00 ± 0.00, respectively (Figure 1; Table 1). In the tissue of proventriculus, which had a scoring system of 5 grades (0-4), the lesions scoring on days 3, 6, and 7 were 1.47 ± 0.24, 2.20, and 2.50 ± 0.00, respectively (Table 2). In the group of caecal tonsil, brain, liver, and lung tissues, which had scoring system of 4 grades (0-3), the scoring in caecal tonsil, on days 3, 6, and 7, was 3.00 ± 0.00, 3.00, 3.00 ± 0.00, respectively (Figure 2; Table 3).

Amidst all collected tissues, only trachea and caecal tonsil obtained full scores. Control group tissues scored 0.

### 3.2. Experiment 2

#### 3.2.1. Appearance of Lesions in Infected Tissues from hr 2 Till Day 6 pi

The lesions appearing in the HE stained tissues were recorded at different times. At hr 2 pi, the lesions appeared in the trachea (2/3). At hr 4 pi, the lesions appeared in the trachea (3/3) and caecal tonsil (1/3). At hr 6 pi, the lesions also appeared in the trachea and caecal tonsil, but in the latter, the number of positive tissues increased to be 3/3. At hr 12 pi, beside the previous positive tissues, some lesions appeared in proventriculus (1/3) and spleen (1/3). On day 1 pi, the appearance level persisted as previously, but the number of positive tissues increased in the proventriculus and spleen to be (3/3). Moreover, some lesions appeared (3/3) in the kidney, and also (1/3) in the bursa of Fabricius, liver, and lung. On day 2 pi, it was different from day 1 pi, the number of positive tissues of liver and lung became 3/3. In the heart and thymus, the lesions appeared (3/3), and intestine (1/3). On day 4 pi, the difference, from previous days, was the appearance of some lesions in the brain (1/3) and more tissues of the intestine and bursa of Fabricius experienced lesions appearance (3/3). On day 5 pi, a single variation was recorded, and it was the appearance of some lesions in the brain tissue in 2/3 (Table 4).

Lesions were not induced in the control group tissues.

#### 3.2.2. Numbers and Percentages of Different vNDV-Infected Tissues Manifesting Lesions

From hr 2 pi till day 6 pi, among 24 infected chickens, different collected tissues were stained with HE and examined; the lesions appeared in 23 out of 24 trachea tissues (95.83%), 19 out of 24 caecal tonsil tissues (79.17%), 13 out of 24 proventriculus tissues (54.17%), 13 out of 24 spleen tissues (54.17%), 12 out of 24 kidney tissues (50.00.00%), 10 out of 24 lung tissues (41.67%), 10 out of 24 liver tissues (41.67%), 9 out of 24 thymus tissues (37.50%), 9 out of 24 heart (37.50%), 8 out of 24 bursa of Fabricius tissues (33.33%), 7 out of 24 intestine tissues (29.17%), and 3 out of 24 brain tissues (12.50%) (Table 5.).

#### 3.2.3. Scoring of Lesions Induced by vNDV on Tissues Stained with HE Since hr 2 Till Day 5 pi

Among the group of trachea, bursa of Fabricius, spleen, and kidney-HE stained tissues which had scoring system composed of 6 grades (0-5), from hr 2 pi till day 5 pi, in the trachea at hr 2 pi, the scoring of 0.20± 0.12 was recorded then it increased gradually to be 2.47 ± 0.18 on day 5 pi. In the spleen at hr 2 pi, scoring of 0.00 ± 0.00 was recorded and then it increased gradually to be 2.73 ± 0.07 on day 5 pi (Table 6). The lesions induced in one tissue were different in type from the lesions induced in other tissues.

Amidst the group of proventriculus, thymus, and heart-HE stained tissues which had a scoring system of 5 grades (0-4), the scorings in proventriculus were detected first at hr 6 pi and scored 0.20± 0.20. Then it increased gradually to be 3.00±0.120 on day 5 pi (Table 7). The lesions induced, in one tissue, were different in type from the lesions induced in other tissues.

Amongst the group of brain, caecal tonsil, liver, intestine, and lung tissues that had scoring system of 4 grades (0-3), in the caecal tonsil, the lesions were detected first at hr 4 pi (0.20 ± 0.20) and then it increased gradually to be 2.13 ± 0.12 on day 5 pi. In the liver, the lesions were detected first on day 1 pi (0.40± 0.40) and then it increased to be 2.13 ± 0.07 on day 5 pi (Table 8). However, the lesions induced in one tissue were different in type from the lesions induced in other tissues.

Control group tissues scored 0.

### 3.3. Experiment 3

In the group of the trachea, bursa of Fabricius, and spleen tissues, which had a scoring system composed of 6 grades (0-5), the lesions induced by vNDV in the trachea, bursa of Fabricius, and spleen scored 5.00 ± 00, 4.84 ± 0.09, and 5.00 ± 00, respectively. The lesions induced by lNDV in the trachea, bursa of Fabricius, and spleen scored 1.40 ± 0.15, 2.28 ± 0.12, 1.80 ± 0.13, respectively. The lesions induced in control tissues were 00 ± 0 (Table 9).

In the group of thymus and proventriculus tissues, which had a scoring system composed of 5 grades (0-4), the lesions induced by vNDV in the thymus and proventriculus scored 4.00 ± 00 and 4.00 ± 00, respectively. The lesions induced by lNDV in the thymus and proventriculus tissues scored 0.20 ± 0.09 and 1.40 ± 0.17, respectively. The lesions induced in the control tissues were 00 ± 0 (Table 10).

In the group of the brain, caecal tonsil, and liver tissues, which had a scoring system composed of 4 grades (0-3), the lesions induced by vNDV in the brain, caecal tonsil, and liver scored 0.12 ± 08, 2.96 ± 0.04, and 3.00 ± 00, respectively. The lesions induced by lNDV in the brain, caecal tonsil, and liver tissues scored 0.36 ± 07, 0.96 ± 16, and 1.16 ± 0.04, respectively. The lesions induced in the control tissues were 00 ± 00 (Table 11).

## 4. Discussion

The differences due to the strains of NDV, with respect to tissue tropism and virulence, are attributed to the presence of cellular proteases. Such enzymes are important for the activation of the viral Fusion (F) glycoprotein precursor F0 [10–12]. When a virulent strain infection occurs, some ubiquitous host proteases split (cleave) F0 protein into subunits F1 and F2. However, these ubiquitous proteases exist in the majority of tissues. Thus, the virulent strains spread at a high pace to many host tissues. On the other hand, in an avirulent strain infection, split (cleavage) of F protein takes place just in the cells that have trypsin-like enzymes. Consequently, the infection is confined to the digestive or respiratory mucosa [11, 13]. The virulent and avirulent strains are chemically differentiated by characterization of the amino acid sequence at the cleavage site of the fusion protein [14].

In a previous study [5], chickens were inoculated with Lasota 1 and Lasota 2 when they were 21 days old and 31 days old, respectively. The tracheal samples were collected when chickens were 40 days old. The lesions were scored in 4 grades (0 -3). In other studies [6, 9], the bursa of Fabricius, thymus, caecal tonsil, and spleen were collected 4 days postinoculation of Quail2006 isolate (velogenic strain) in commercial 5 weeks old broiler chickens, whereas the bursa of Fabricius, thymus, Harderian gland, caecal tonsil, bone marrow, and spleen were collected 4 days postinoculation of LaSota (lentogenic strain) in another group of 5-week-old broiler chickens. The lesions in the bursa of Fabricius were scored in 6 grades (0-5), in the thymus and spleen, the lesions were scored in 5 grades (0–4), in the caecal tonsil and bone marrow, the scoring was carried out in 4 grades (0-3), and in Harderian gland, they were 3 grades (0–2). In brief, it could be summarized that in previous studies, the scoring, of the lesions, was carried out for a maximum of 6 different tissues. The scoring ranges were either 0–2 or 0–3 or 0–4 or 0-5. On the other hand, in the current study, a system was designed for scoring of the lesions induced by different strains in 15 different tissues. The system consisted of 4-6 grades.

The lesions induced in the trachea tissue by AF2240 isolate (velogenic strain) in Experiments 1, 2, and 3 obtained full scores (5.00 ± 0.00) twice. In contrast, the lesions induced by V4 isolate (lentogenic strain) in experiment 3 and the lesion induced by Lasota (lentogenic strain), in the study [5], obtained a scoring below 2. The lesions induced in the bursa of Fabricius and spleen tissues by AF2240 isolate (velogenic strain) in Experiments 1, 2, and 3 obtained a scoring of 4 or above twice, while the lesions induced in the bursa of Fabricius and spleen by V4 isolate (lentogenic strain) in experiment 3 and the lesions induced by Lasota (lentogenic strain), in the study [5], obtained a scoring below 3 and 2, respectively.

The lesions induced in the thymus tissue by AF2240 isolate (velogenic strain) in Experiment 3 obtained full scores (4.00 ± 0.00). However, Quail2006 isolate (velogenic strain), in study [5], induced lesions which had scoring just slightly below 4.00, whereas the lesions induced by V4 isolate (lentogenic strain) in experiment 3 and the lesions induced by Lasota (lentogenic strain), in study [5], obtained a scoring below 2. The lesions induced in the caecal tonsil tissue by AF2240 isolate (velogenic strain) in Experiments 1, 2, and 3 obtained full scores (3.00 ± 0.00) twice. In contrast, the lesions induced by V4 isolate (lentogenic strain) in experiment 3 and the lesion induced by Lasota (lentogenic strain), in study [5], obtained a scoring below 2.

The findings of experiment 2 were helpful in understanding some points of pathogenesis and tissue tropism. It seemed that the virus was transported from the nasal cavity to the trachea by the respiratory tract lumen direct contact. Then probably it reached the caecal tonsil, proventriculus, spleen, and the rest of tissues via viraemia.

The more early tissues were collected postinoculation, the lower lesion scoring was recorded. While the later tissues collected postinoculation, the higher lesion scoring was recorded. Both LaSota isolate and V4 isolate are commercially available vaccines. However, it seemed that LaSota had a milder influence in the trachea and spleen, while V4 induced a milder picture in the caecal tonsil and thymus. It seemed that there was no difference between lentogenic strains in the consequent pictures of the bursa of Fabricius. Further studies should take over the amino acid sequence and nucleotide sequence at the cleavage site of the fusion protein and the fusion gene of different isolates.

## 5. Conclusions

A scoring system was designed. The lesions induced by different strains of NDV in 15 different tissues were scored. The scoring system consisted of 4-6 grades to help evaluate the disease severity and hence to evaluate the strain virulence.

## Figures and Tables

**Table 1:** Lesions scoring (0-5) induced by vNDV in HE stained trachea, bursa of Fabricius, and spleen tissues on days 3, 6, and 7 pi.

	Trachea	Bursa of Fabricius	Spleen
Day 3 pi	5.00 ± 0.00 (3)	2.60 ± 0.12 (3)	3.53± 0.18 (3)
Day 6^∗^ pi (one mortality case)	5.00 (1)	4.40 (1)	4.40 (1)
Day 7 pi	5.00 ± 0.00 (2)	4.50± 0.10 (2)	4.60± 0.00 (2)

*Note.* Scoring was expressed as mean ± SE. Values in brackets indicate the number of chickens.

^∗^One chicken died on day 6 pi. Tissues collected from the control group scored 0.

**Table 2:** Lesions scoring (0-4) induced by vNDV in HE stained proventriculus tissue on days 3, 6, and 7 pi.

	Proventriculus
Day 3 pi	1.47 ± 0.24 (3)
Day 6^∗^ pi (one mortality case)	2.20 (1)
Day 7 pi	2.50± 0.00 (2)

*Note.* Scoring was expressed as mean ± SE. Values in brackets indicate the number of chickens.

^∗^One chicken died on day 6 pi. Tissues collected from the control group scored 0.

**Table 3:** Lesions scoring (0-3) induced by vNDV in HE stained caecal tonsil, brain, liver, and lung tissues on days 3, 6, and 7 pi.

	Caecal Tonsil	Brain	Liver	Lung
Day 3 pi	3.00 ± 0.00 (3)	0.00 ± 0.00 (3)	0.73 ± 0.07 (3)	0.87± 0.07 (3)
Day 6^∗^ pi (one mortality case)	3.00 (1)	0.20 (1)	1.60 (1)	2.20 (1)
Day 7 pi	3.00 ± 0.00 (2)	1.30 ± 0.10 (2)	1.70 ± 0.10 (2)	2.60 ± 0.00 (2)

*Note.* Scoring was expressed as mean ± SE. Values in brackets indicate the number of chickens. ^∗^One chicken died on day 6 pi. Tissues collected from the control group scored 0.

**Table 4:** Appearance of lesions induced by vNDV in different HE-stained tissues from hr 2 till day 6 pi.

Intranasal inoculation of v NDV	Number of tissues in which lesions appeared, out of 3 examined tissues stained with HE
Hour 2 pi	(2/3) in trachea.

Hour 4 pi	(3/3) in the trachea and (1/3) in the caecal tonsil.

Hour 6 pi	(3/3) in the trachea and caecal tonsil.

Hour 12 pi	(3/3) trachea and caecal tonsil, (1/3) in proventriculus and spleen.

Day 1 pi	(3/3) trachea and caecal tonsil, proventriculus, spleen, (1/3) bursa of Fabricius and liver, (3/3) kidney and intestine, and (1/3) in the lung.

Day 2 pi	(3/3) in the trachea, and caecal tonsil, (3/3) proventriculus and spleen, (1/3) in bursa of Fabricius, (3/3) in the liver, kidney, heart, and thymus, (1/3) in the intestine, and (3/3) in the lung.

Day 4 pi	(3/3) in the trachea, and caecal tonsil, (1/3) in the brain, (3/3) in proventriculus, spleen, bursa of Fabricius, liver, kidney, heart, thymus, intestine, and lung.

Day 5 pi	(3/3) in the trachea, caecal tonsil, (2/3) in the brain, (3/3) in proventriculus, spleen, bursa of Fabricius, liver, kidney, heart, and thymus, intestine and the lung.

*Note.* Values between brackets before slash indicate the number of a certain tissue in which lesions were detected pi of vNDV in SPF chickens, while values after slash indicate the whole number of a certain tissue collected, stained with HE, and examined. Lesions were not detected in tissues of control group chickens.

**(a):** 

	Trachea	Caecal Tonsil	Provent-riculus	Spleen	Kidney	Lung
No. of positive tissues^∗^	23 (24)	19 (24)	13 (24)	13 (24)	12 (24)	10 (24)
Percentage of positive tissues^∗^	95.83 %	79.167%	54.17%	54.167%	50.00%	41.67%

**(b):** 

	Liver	Thymus	Heart	Bursa of Fabricius	Intestine	Brain
No. of positive tissues^∗^	10 (24)	9 (24)	9 (24)	8 (24)	7 (24)	3 (24)
Percentage of positive tissues^∗^	41.67%	37.50%	37.50%	33.33%	29.17%	12.50%

*Note.* Values in brackets represent the whole number of SPF chickens which were infected with vNDV from hour 2 pi till day 6 pi. Positive tissue *∗* indicates tissue in which one lesion or more were detected. Lesions were not detected in tissues of control group chickens.

**Table 6:** Lesion scoring (0-5) induced by vNDV in HE stained tissues of the trachea, bursa of Fabricius, spleen, and kidney collected from hr 2 pi till day 5 pi.

	Hr 2 pi	Hr 4 pi	Hr 6 pi	Hr 12 pi	Day 1 pi	Day 2 pi	Day 4 pi	Day 5 pi
Trachea	0.20± 0.12(3)	1.13± 0.07(3)	1.13± 0.07(3)	1.27± 0.07(3)	1.40± 0.12(3)	1.80± 0.12(3)	2.30± 0.18(3)	2.47± 0.18(3)
Bursa of Fabricius	00± 00(3)	0.00± 0.00(3)	0.07± 0.07(3)	0.13± 0.13(3)	0.33± 0.18(3)	1.67± 0.87(3)	1.73± 0.84(3)	1.93± 0.07(3)
Spleen	00± 00(3)	0.13± 0.13(3)	0.20± 0.20(3)	0.20± 0.20(3)	1.07± 0.18(3)	1.67± 0.18(3)	2.53± 0.07(3)	2.73± 0.07(3)
Kidney	00± 00(3)	00± 00(3)	00± 00(3)	0.20± 0.20(3)	0.27± 0.27(3)	0.93± 0.13(3)	2.20± 0.12(3)	2.33± 0.07(3)

*Note.* Values in brackets represent the number of chickens. Hr indicates hour.pi indicates postinoculation of the virus. Control group tissues scored 0.

**Table 7:** Lesion scoring (0-4) induced by vNDV in HE stained tissues of proventriculus, thymus, and heart collected from hr 2 till day 5 pi.

	Hr 2 pi	Hr 4 pi	Hr 6 pi	Hr 12 pi	Day 1 pi	Day 2 pi	Day 4 pi	Day 5 pi
Proventri-culus	00± 00(3)	00± 00(3)	00± 00(3)	0.20±0.20(3)	2.20± 0.12(3)	2.20±0.12(3)	2.27± 0.07 (3)	3.00± 0.12(3)
Thymus	00± 00(3)	00± 00(3)	00± 00(3)	00± 00(3)	00± 00(3)	1.73±0.07(3)	2.33± 0.07(3)	2.73±0.07 (3)
Heart	00± 00(3)	00± 00(3)	00± 00(3)	00± 00(3)	00± 00(3)	0.40±0.12(3)	2.40± 0.12(3)	2.47± 0.07(3)

*Note.* Values in brackets represent the number of chickens. Hr indicates hour. pi indicates postinoculation of the virus. Control group tissues scored 0.

**Table 8:** Lesion scoring (0-3) induced by vNDV in HE stained tissues of proventriculus, thymus, and heart collected from hr 2 till day 5 pi.

	Hr 2 pi	Hr 4 pi	Hr 6 pi	Hr 12 pi	Day 1 pi	Day 2 pi	Day 4 pi	Day 5 pi
Brain	00± 00(3)	00± 00(3)	0.00±0.00(3)	0.00±0.00(3)	00± 00(3)	0.00± 0.00(3)	0.33± 0.33(3)	0.60± 0.31(3)
Caecal tonsil	00± 00(3)	0.20± 0.20(3)	0.47±0.07(3)	1.33±0.07(3)	1.20± 0.12(3)	2.00± 0.12(3)	2.00± 0.07(3)	2.13± 0.12(3)
Liver	00± 00(3)	00± 00(3)	00± 00(3)	00± 00(3)	0.40±0.40(3)	0.53± 0.07(3)	2.13± 0.07 (3)	2.13± 0.07(3)
Intestine	00± 00(3)	00± 00(3)	00± 00(3)	00± 00 (3)	00± 00(3)	0.07± 0.07(3)	2.00± 0.12(3)	2.27± 0.07(3)
Lung	00± 00(3)	00± 00(3)	00± 00(3)	00± 00(3)	0.60± 0.60(3)	1.47± 0.07(3)	2.07± 0.07(3)	2.33± 0.07(3)

*Note.* Values in brackets represent the number of chickens. Hr indicates hour. pi indicates postinoculation of the virus. Control group tissues scored 0.

**Table 9:** Lesions scoring (0-5) induced by velogenic and lentogenic NDV strains in HE stained trachea, bursa of Fabricius, and spleen tissues on day 5 pi.

	vNDV	lNDV	Control
Trachea	5.00 ± 00 (5)	1.40 ± 0.15 (5) 0.20 ± 0.11^*∗*a^	00 ± 00 (5)
Bursa of Fabricius	4.84 ± 0.09 (5) 5.00^∗∗^	2.28 ± 0.12(5) 2.30^*∗*b^	00 ± 00 (5)
Spleen	5.00 ± 00 (5) 4.00^∗∗^	1.80 ± 0.13 (5) 1.30^*∗*b^	00 ± 00 (5)

Values between brackets indicate the number of examined tissues.

^*∗*a^Lesion scoring of tissues inoculated with LaSota 1 and 2 when chickens were 21 days old and 31 days old, respectively. Lesions were recorded when chickens were 40 days old [5].

^*∗*b^Lesion scoring of tissues inoculated with LaSota (GenBank accession numberAF077761). Lesions were recorded 4 days pi [6]. ^∗∗^Lesion scoring of tissues infected by Quail2006 isolate, velogenic strain, lesions were recorded 4 days postinoculation, chicken embryo mean death time (MDT) = 47.0, intracerebral pathogenicity index (ICPI) = 1.94, GenBank accession number EU518684 [9].

**Table 10:** Lesions scoring (0-4) induced by velogenic and lentogenic NDV strains in HE stained thymus, proventriculus, and Harderian gland tissues on day 5 pi.

	*vNDV*	*lNDV*	*Control*
Thymus	4.00 ± 00 (5) 3.70^∗∗^	0.20 ± 0.09 (5) 1.30^*∗*b^	00 ± 00 (5)
Proventriculus	4.00 ± 00 (5)	1.40 ± 0.17 (5)	00 ± 00 (5)
Harderian gland^*∗*c^	NA	1.30^*∗*b^	00 ± 00 (3)

Values between brackets indicate the number of examined tissues.

^*∗*a^Lesion scoring of tissues inoculated with LaSota 1 and 2 when chickens were 21 days old and 31 days old, respectively. Lesions were recorded when chickens were 40 days old [5].

^*∗*b^Lesion scoring of tissues inoculated with LaSota (GenBank accession number AF077761). Lesions were recorded 4 days postinoculation [6].

^∗∗^Lesion scoring of tissues infected by Quail2006 isolate, isolate's name includes avian species and year of isolation, velogenic strain, and lesions were recorded 4 days postinoculation, chicken embryo mean death time (MDT) = 47.0, intracerebral pathogenicity index (ICPI) = 1.94, GenBank accession number EU518684 [9].

^*∗*c^Harderian gland tissue was scored according to a system composed of 3 grades only.

NA = Tissues were not collected.

**Table 11:** Lesions scoring induced by velogenic and lentogenic NDV strains in HE stained brain, caecal tonsil, liver, and bone marrow tissues on day 5 pi.

	vNDV	lNDV	Control
Brain	0.12 ± 08 (5)	0.36 ± 07 (5)	00 ± 00 (5)
Caecal tonsil	2.96 ± 0.04 (5) 3.00^∗∗^	0.96 ± 16 (5) 1.70^*∗*b^	00 ± 00 (5)
Liver	3.00 ± 00 (5)	1.16 ± 0.04 (5)	00 ± 00 (5)
Bone marrow	NA	3.00^*∗*b^	00 ± 00 (3)

Values between brackets indicate the number of examined tissues.

^*∗*a^Lesion scoring of tissues inoculated with LaSota 1 and 2 when chickens were 21 days old and 31 days old, respectively. Lesions were recorded when chickens were 40 days old [5].

^*∗*b^Lesion scoring of tissues inoculated with LaSota (GenBank accession numberAF077761). Lesions were recorded 4 days pi [6].

^∗∗^Lesion scoring of tissues infected by Quail2006 isolate, isolate's name includes avian species and year of isolation, velogenic strain, and lesions were recorded 4 days pi, chicken embryo mean death time (MDT) = 47.0, intra-cerebral pathogenicity index (ICPI) = 1.94, GenBank accession number EU518684, [9].

NA = Tissues were not collected.

## Data Availability

The data used to support the findings of this study are included within the article.
